# Characterizing the escalation of pyrethroid resistance and its impact on bed nets efficacy alongside molecular basis in *Anopheles funestus* from Cameroon

**DOI:** 10.1186/s12936-025-05542-z

**Published:** 2025-09-30

**Authors:** Hervé Raoul Tazokong, Stevia Ntadoun Tchamga, Magellan Tchouakui, Tatiane Assatse, Steve Valdi Djova, Leon M. J. Mugenzi, Gadji Mahamat, Onana Boyomo, Charles Sinclair Wondji

**Affiliations:** 1 Medical Entomology Department, Centre for Research in Infectious Diseases (CRID), Yaoundé, Cameroon; 2 Department of Microbiology, Faculty of Science, University of Yaoundé 1, Yaoundé, Cameroon; 3 Department of Biochemistry, Faculty of Science, University of Yaoundé 1, Yaoundé, Cameroon; 4 Department of Animal Biology, Faculty of Science, University of Yaoundé 1, Yaoundé, Cameroon; 5 Vector Biology Department, Liverpool School of Tropical Medicine, Pembroke Place, Liverpool, L3 5QA UK

**Keywords:** Malaria, Resistance escalation, *Anopheles funestus*, Pyrethroids, Cameroon, LLINs

## Abstract

**Background:**

Malaria prevention still relies greatly on vector control interventions. However, increasing levels of resistance to pyrethroids across Africa have significantly reduced the efficacy of pyrethroid-based interventions leading to an increase of malaria burden. Consequently, it is imperative to characterize the extent and molecular basis of this resistance.

**Methods:**

This study was conducted from 2020 to 2021 in a South-North transect across Cameroon. WHO tube assay was used to assess the susceptibility profile of *Anopheles funestus* to the four main classes of insecticides. The efficacy of bed nets was evaluated using cone assay. Known genetic resistance markers and gene expression were determined using PCR and quantitative PCR techniques, respectively. Taqman assay and nested polymerase chain reaction (PCR) were used to determine *Plasmodium* sporozoite infection.

**Results:**

High pyrethroid resistance intensity was noticed in all sites with mortalities ranging from 80–93.9%, 84.9–96.7% and 82% for permethrin, deltamethrin and alphacypermethrin at 10 × concentration respectively. This high level of resistance led to dramatic inefficacy of pyrethroid-only nets with 0–17% mortality recorded 24-h post exposure while PBO-based nets displayed optimal efficacy. Sporozoite infection rates ranged from 0–16.5% across the study sites. However, there was no clear relationship between the infection rate and the intensity of pyrethroid resistance. The L119F-*GSTe2* allele was higher in the South (56–68%) compared to the North (20–37%) meanwhile the P450-linked 4.3 kb structural variant was fixed contrasting with the absence of the *CYP6P9a/b-R*, 6.5 kb insertion and N485I-*Ace1* alleles. Furthermore, the L119F-*GSTe2* allele confers significant ability to mosquito to survive permethrin. In addition, the *CYP325A, CYP6P5, CYP6P9a/b, and* the *Carb2514* were the most overexpressed genes in pyrethroid resistant mosquitoes. However, no further association was noticed between these alleles/genes and increasing doses of pyrethroids.

**Conclusion:**

This study confirms the escalation of pyrethroid resistance across Cameroon and the inefficacy of pyrethroid-only nets and highlights genes potentially implicated in the aggravation of insecticide resistance with implications on vector control strategies.

## Background

Malaria remains a global public health concern with 249 million cases and 608 000 deaths recorded in 2022, with pregnant women and children being the most affected [1]. Disease control relies heavily on insecticide-based interventions, notably long-lasting insecticidal nets (LLINs) and indoor residual spraying (IRS), both contributing the most to the decrease in malaria burden since 2000 [1, 2]. However, efforts to reduce malaria burden have stalled recently due to several challenges among which the growing reports of resistance to insecticides in major malaria vectors. With the World Health Organization (WHO) advocating for the implementation of a subnational tailoring control approach, it is crucial that data on resistance be generated nation-widely to implement evidence-based interventions [1]. Several malaria-endemic countries have reported resistance to the four main classes of insecticides and 27 countries noticed evidences of intensification of resistance to pyrethroids, the main ingredient of bed nets [1]. For example, an increase of pyrethroid resistance has been reported in both *Anopheles gambiae *sensu lato (*s.l.*) and *Anopheles funestus* from Uganda [3], Ghana [4] and Malawi [5], Democratic Republic of Congo [6], as well as reduced efficacy of pyrethroid and PBO (piperonyl butoxide)-based nets [7–12]. This situation highlights the challenge faced by national malaria control programmes in sustaining decades of successes over malaria through current insecticide-based interventions.

Cameroon is among the eleven countries contributing to over 70% of the global malaria burden and has registered 4121 deaths in 2022 [1, 13]. Bed nets coverage is 81.8% and *An. gambiae*, *Anopheles coluzzii*, and *An. funestus* are the major malaria vectors [14]. The trend of susceptibility profile shows high level of pyrethroid resistance in *An. coluzzii* [15] and *An. gambiae* [16–20] with the identified mechanism supporting this strong resistance being target site mutation, cytochrome P450s, Glutathione S-Transferases (GSTs) and cuticular proteins [15–19]. However, unlike *An. gambiae s.l.* which has been extensively characterized, no country-wide characterization of resistance has been performed for *An. funestus*. Resistance to pyrethroids has been noticed in *An. funestus* from Central and Northern Cameroon with association from the L119F-*GSTe2* marker [16, 21–23] in addition to the up-regulation of the *GSTe2* [24] and some P450s genes including *CYP6P5, CYP6P9b*/a and *CYP325A* [16, 25]. There are evidences that genetic resistance markers may exacerbate malaria transmission by increasing the infection rate or the entomological inoculation rate [26, 27]. For example, in *An. funestus,* the L119F-*GSTe2* has been associated to high *Plasmodium* infection rate with homozygous resistant individual mosquito being more infected that their counterparts homozygous susceptible [26]. In contrast, an inverse correlation was observed between the resistance marker 4.3 structural variant (SV) and malaria parasite infection [28]. Findings from laboratory selected *An. gambiae* colony experimentally infected with *Plasmodium falciparum* showed increased oocyst and sporozoite infection rates in resistant mosquito compared to susceptible one [29]. These results show how phenotypic resistance or genetic markers can impact malaria parasite infection rate which could result to increase malaria transmission. With the increasing level of pyrethroids resistance reported in the principal malaria vectors, it remains unknow to which extent the L119F-*GSTe2* combined with other known genetic markers and the gene expression could impact the ability of the *An. funestus* population to survive high doses of pyrethroid. Therefore, a large scale and comprehensive entomological study was conducted on this vector to assess its geographic resistance pattern and impact on bed nets efficacy in four eco-geographical locations from Cameroon. This includes the distribution of known genetic makers, sporozoite infection, the gene expression and resistance profiles, thereby providing relevant information to malaria control programme in the deployment of current and future insecticide-based interventions. Findings revealed the predominance of *An. funestus *sensu stricto (*s.s.*) as the principal malaria vectors in the study areas. This species exhibited high resistance intensity to pyrethroid leading to dramatic loss in efficacy of pyrethroid-only nets while PBO-based nets remained effective.

## Methods

### Study site

**Fig. 1:**
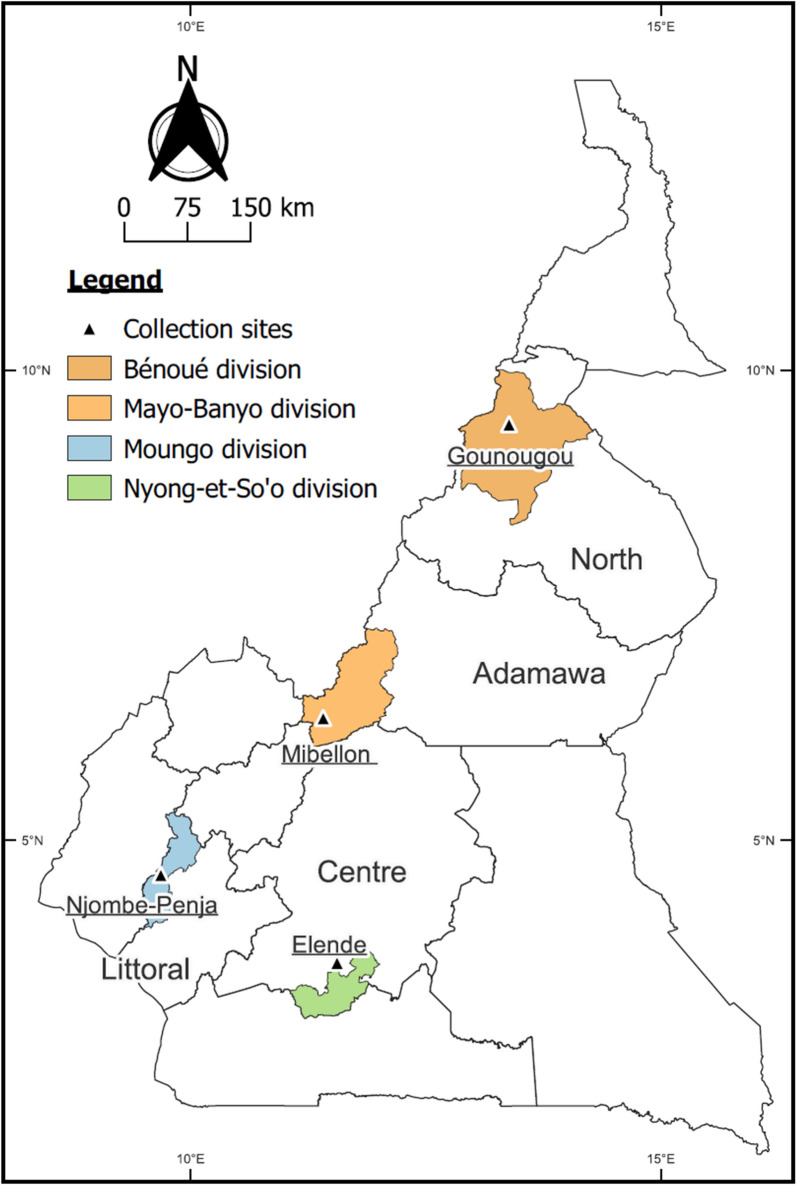
Map of Cameroon showing mosquito collection sites

The study was conducted from August 2020 to December 2021 in four eco-geographical locations belonging to the south (Elende: 3°41′57.27''N, 11°33′28.46''E and Njombe-Penja: 04°34’N, 09°39’E) to north (Mibellon: 6°46′N, 11°70′E and Gounougou: 9°03′00″N, 13°43′59″E) transect in Cameroon (Fig. 1). Each of this study site has a unique ecological profile influencing mosquito breeding, insecticide exposure and selection pressure. Elende is a peri-urban area near to Yaoundé Nsimalen International Airport, Nkolmetet subdivision, Centre region, characterized by high humidity (65–80%) and an average annual rainfall of 1800 mm. Its proximity to the Mefou River and numerous marshy areas creates ideal breeding sites for *An. funestus*. Road construction and deforestation also create temporary and permanent sites suitable for *An. gambiae*. Agriculture is the main human activity, with crops like cassava and vegetables [12]. Pyrethroid insecticides are heavily used for crop protection, often sprayed at least five times per campaign especially in tomatoes and watermelon fields. The majority of houses are built with brick or mud walls, and are topped with either iron sheets or thatched roofs. Public health interventions primarily rely on pyrethroid-only bed nets, specifically Olyset and PermaNet 2.0, which have a high coverage of approximately 70% [30].

Njombe-Penja is a peri-urban area of the Moungo division having tropical climate with high rainfall, known for its extensive industrial banana plantation. The plantation utilizes intensive, year-round spraying of neonicotinoid and pyrethroid insecticide mixtures by planes. Other crops, such as peppercorn, pineapple, and cocoa, are also protected using these mixtures, and to a lesser extent, carbamates and organophosphates. The majority of houses feature plank walls and sheet metal roofs. Mosquito vector control is limited, with household bed net ownership and usage estimated at only 30% as noticed during the sample collection.

Mibelllon is a rural village in the Bankim subdivision, Adamawa region, situated in a transitional zone between forest and savannah. Permanent swamps and a lake provide abundant mosquito breeding sites near households. The village relies on farming, hunting, and fishing. Farmers use a high level of insecticides from three classes: carbamates, neonicotinoids, and pyrethroids particularly in watermelon and coffee farms [22]. Most houses are built with mud and brick walls and have thatched or iron sheet roofs. The primary vector control tool is the long-lasting insecticidal treated nets PermaNet 2.0, distributed during a mass campaign in 2015. Gounougou is a village in the Lagdo subdivision, North region, surrounded by the Bénoué River. The climate is dry, with an average annual rainfall of 900–1000 mm. The river and its dam provide irrigation for vast rice fields (≈15,000 hectares), which serve as prime breeding sites for both *An. funestus* and *An. gambiae*. *Anopheles funestus s.l.* is dominant during the dry season when temporal breeding sites for *An. gambiae* are absent [23]. Rice is cultivated twice annually, with insecticides sprayed several times per campaign. Historically, insecticides like endosulfan, profenofos, and cypermethrin were used on cotton, while pyrethroids have been the main treatment for rice crops [31]. Almost all houses feature brick walls with thatched roofs. Pyrethroid-only nets were distributed in mass campaigns in 2019, serving as the main vector control tool.

### Mosquito collection and identification

After obtaining household consent, blood-fed female mosquitoes resting indoor were collected using electrical aspirator Prokopack technique (John W. Hock co., USA) from 6:00 to 10:00 am. At each site, 20 to 30 households randomly selected were screened during 6–7 days with an average of 35 mosquitoes collected per household/day. Female mosquitoes were fed with 10% of sugar for 4–5 days and forced to lay eggs in individual Eppendorf tubes then eggs were maintained in paper cups to allow them to hatch and larvae were transferred in plastic bowls for rearing [32]. All collected mosquitoes were identified morphologically [33], but only those from the *An. funestus* group were retained. This was because *An. funestus s.l.* was the dominant species, representing over 90% of the total vector population, except in Gounougou. Mosquitoes head/thorax and abdomen regardless of egg-laying success were separated and subjected to DNA extraction using the Livak protocol [34]. The DNA extracts were used for molecular identification [35].

### Assessment of *Plasmodium* sporozoite infection rate

The head/thorax of mosquitoes (oviposited and non-oviposited) were tested for the presence of sporozoites using the enzyme-linked immunosorbent assay (ELISA) in Elende, as described previously [36]. While in the other locations, Taqman assay was used to screen all the four *Plasmodium* species: *P. falciparum* and OVM (*Plasmodium ovale, Plasmodium vivax, Plasmodium malariae*) [37] then positive sample was validated by nested-PCR [38].

### Insecticide susceptibility assay and synergist-insecticide bioassay

The resistance profile of 3–5 days old F_1_ *An. funestus* was established following WHO protocol [39] using diagnostic concentration (DC) of the following insecticides: (i) pyrethroids type I permethrin (0.75%) and type II deltamethrin (0.05%) and alpha-cypermethrin (0.05%); (ii) the carbamates propoxur (0.1%) and bendiocarb (0.1%); (iii) the organophosphate malathion (5%) and pirimiphos-methyl (0.25%); and the organochlorine DDT (4%). For each insecticide, a total of 100 mosquitoes were exposed from four replicates of 25 mosquitoes each. A negative control was used by exposing mosquito to untreated paper. The mortality was measured 24 h after exposure and resistance assessed according to WHO guidelines [39].

Synergist assay was conducted using piperonyl butoxide (PBO) following the WHO guidelines [39]. Briefly, four replicates of 20–25 mosquitoes per replicate were pre-exposed to PBO alone for 1 h then transferred into tubes containing insecticide treated paper permethrin (0.75%), deltamethrin (0.05%) and alpha-cypermethrin (0.05%) for an additional 1 h of exposure [39]. Mortality was recorded 24 h post-exposure. Two controls were used: mosquitoes exposed to PBO alone without subsequent exposure to insecticides and those exposed to untreated paper. To assess the potential involvement of P450-based metabolic resistance, comparison of the 24 h mortality between pre-exposed PBO and insecticide alone [39] was performed.

### Quantification of resistance intensity

The strength of pyrethroid resistance was determined by additional bioassays with 5 × and 10 × DCs of permethrin (3.75% and 7.5%), deltamethrin (0.25% and 0.5%) and alpha-cypermethrin (0.25% and 0.5%) following the WHO procedure [39]. Interpretation of the results was done accordingly [39]. Dead and alive mosquitoes were kept in Silica gel and RNA*later*^®^ (Thermo FisherScientific, Waltham, MA, United States) respectively and stored at − 80 °C until the molecular analyses.

### Assessing the bio-efficacy of bed nets using cone assay

Cone assay [40] was conducted by exposing 5 replicates of 10 mosquito each to determine the bio-efficacy of the following bed nets: Olyset^®^ (permethrin 2%), Olyset^®^ Plus (permethrin 2% plus 1% of PBO), PermaNet® 2.0 (deltamethrin 1.4–1.8 g/kg ± 25%), PermaNet^®^ 3.0 [(both the side panel (deltamethrin 2.1–2.8 g/kg ± 25%) and the top (4.0 g/kg ± 25% plus PBO 25 g/kg ± 25%)], Interceptor^®^ (200 mg/m^2^ ± 25% alpha-cypermethrin), Duranet^®^ (250 mg/m^2^ alpha-cypermethrin) and Royal Guard (225 ± 56.5 mg/m^2^ for both alpha-cypermethrin and pyriproxyfen).

### Genotyping of resistance markers

Known genetic resistance markers were genotyped in 35–50 individual field mosquitoes (oviposited and non-oviposited females) randomly chosen per location to determine pattern of geographical distribution and subsequently progenies from egg-laid mosquitoes were used for genotype/phenotype associations. This includes: the L119F-*GSTe2* (Glutathione S-Transferase epsilon 2) known to confer resistance to DDT and pyrethroid was genotyped using the allele specific PCR [26]; the *CYP6P9a-R* and *CYP6P9b-R* alleles conferring pyrethroids resistance were amplified as described previously [41, 42]. The structural variants (SV) 6.5 kilobase (kb) [43] and 4.3 kb [28], involved in pyrethroid resistance were genotyped using a multiplex PCR and the TaqMan assay for the N485I-*ace1* involved in bendiocarb resistance [44]. The molecular marker which was either fixed or absent was excluded for genotype/phenotype association and only the L119F-*GSTe2* was used to make a corelation between 35 dead and 35 alive mosquitoes after exposure to 1x, 5 × and 10 × DCs. A pairwise comparison of genotypes and the allele frequency was performed among dead/alive for each insecticide at each concentration.

### Transcription profile of detoxification genes using real time quantitative PCR

Quantitative reverse transcription PCR (qRT-PCR) was used to assess the expression level of the following genes: P450s *CYP325A, CYP9K1, CYP6P5, CYP6Z1, CYP6Z3, CYP6AA1, CYP6P4a, CYP6P9a and b, CYP6M7* and *CYP6P2*), esterase (*carb2514; AFUN002514*) previously involved in pyrethroid and GST (*GSTe2* involved in pyrethroid and DDT resistance). RNA (Ribonucleic acid) extraction was performed using Picopure RNA Isolation Kit (Arcturus) on three pools of 10 mosquitoes each from survivors at 1x, 5 × and 10 × DC to both permethrin and alphacypermethrin, unexposed (control) from Mibellon. Whereas in Gounougou, this was done on mosquito surviving deltamethrin 1 × and unexposed plus the susceptible strain FANG. The extracted RNA was used as template for cDNA synthesis using the superscript III (Invitrogen) with oligo-dT20 and RNase H, following the manufacturer’s instructions. The relative expression level and fold-change (FC) was calculated individually compared to the reference susceptible strain Fang according the 2^−ΔΔCT^ method [45] after normalisation with two housekeeping genes *actin* and *RSP7*.

### Statistical analyses

Chi square test were used to assess the recovery in susceptibility by comparing mortality rates between PBO and non PBO exposure. Statistical analysis was performed using GraphPad Prism 8.0 (GraphPad Inc., La Jolla, CA, USA) with significance at p < 0.05 (Confidence interval: CI at 95%). Unpaired Student’s t-test was used to determine the level of significance in gene expression compared to FANG and within phenotypes (unexposed, 1x, 5 × and 10x). Fisher’s exact test was used to determine whether any difference in proportion observed for the genotype/allele contingency table is significant and odds ratio (OR) was used to quantify the strength of association between the *GSTe2* genotype/allele in alive/dead mosquitoes at 1x, 5 × and 10 × DCs for both permethrin and alphacypermethrin.

## Results

### Species composition

**Fig. 2:**
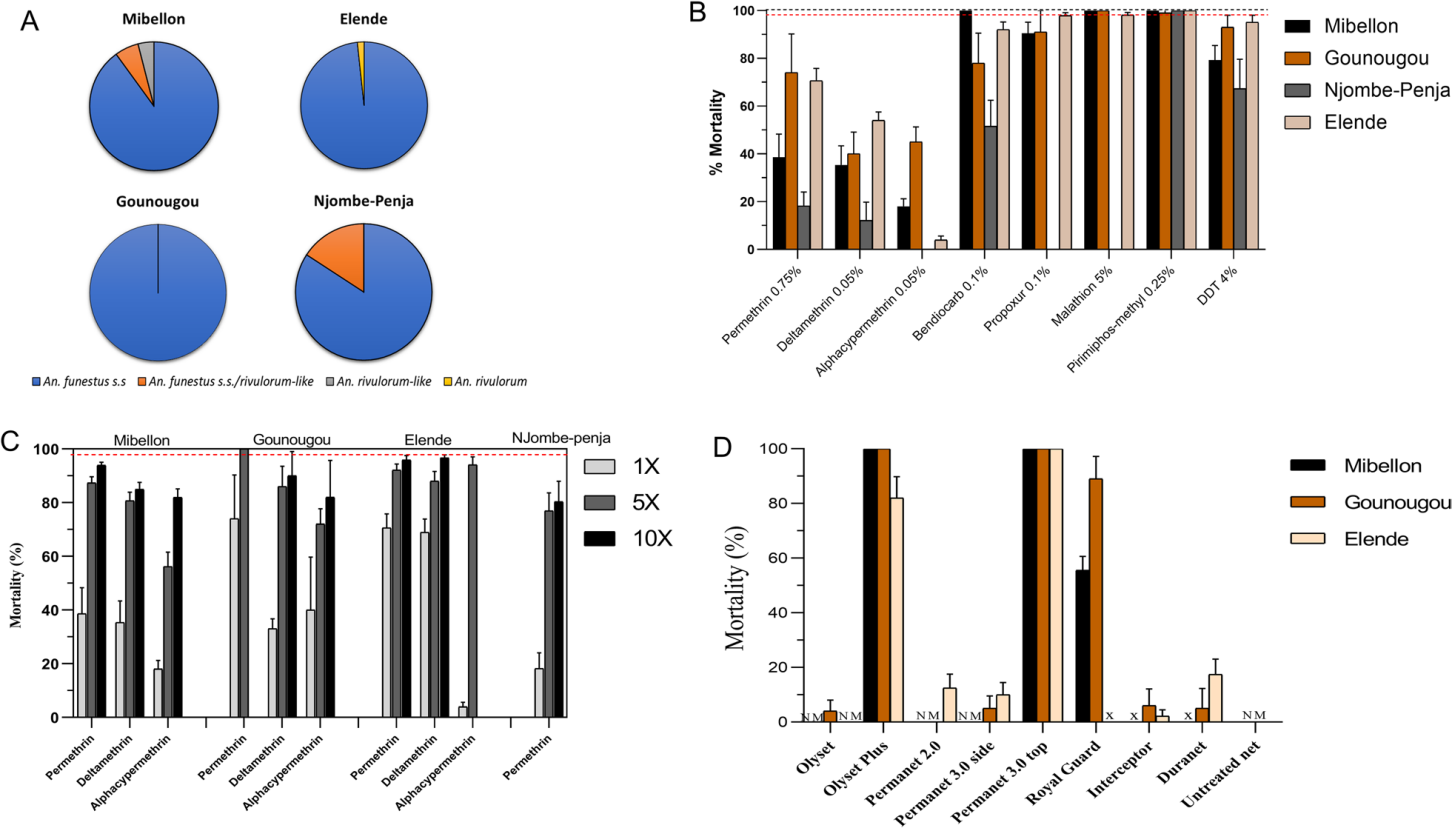
Species composition (**A**). Susceptibility profile of F_1_ mosquito across the four studied sites (**B**) recorded mortality 24 h post-exposure of *An. funestus* s.l. to the diagnostic concentration and (**C**) Intensity of pyrethroid resistance. Data are shown as mean ± standard mean error (SEM), red dot line indicate threshold of 98% mortality, x: insecticide not tested in this site. **D** Bioefficacy of pyrethroid-only nets and PBO-based nets against *An. funestus s.l.,* recorded mortality after 24 h post-exposure. NM = no mortality, x = net not tested in this site

Morphological identification revealed that *An. funestus s.l..* accounted for over 90% of all mosquitoes collected in all locations, except in Gounougou, where *An. funestus s.l.* and *An. gambiae s.l.* represented 64% and 34%, respectively. Molecular identification revealed that *An. funestus *sensu stricto (*s.s.)* was the dominant species among the funestus group in all localities with the following frequencies: 100% (60/60) in Gounougou, 98.3% (59/60) in Elende, 90% (45/50) in Mibellon and 84.2% Njombe-Penja (Fig. 2A). Other species identified were; *Anopheles rivulorum*-like and *An. rivulorum* only present in Mibellon and Elende at 4% and 1.7%, respectively, and hybrid of *An. funestus s.s./rivulorum*-like (15.8% and 6% in Njombe-penja and Mibellon, respectively).

### Sporozoite infection rates

While no infection was detected in mosquito from both Gounougou (0/127) and Njombe (0/92), the sporozoite infection rate was 6.5% (6/92) in Mibellon and 16.5% (14/85) in Elende using TaqMan and ELISA techniques, respectively. In Mibellon, out of 6 positive samples from TaqMan, 5 were confirmed as *P. falciparum* with nested-PCR*.*

### Susceptibility profile

Resistance to all pyrethroids tested at the diagnostic concentration (1x) was observed in mosquito populations from every location. Mortality rates varied, ranging from 18 to 70% for permethrin, 12–54% for deltamethrin, and 3–45% for alphacypermethrin (Fig. 2B). The Njombe-Penja population showed significantly lower mortality to permethrin (18.2%) and deltamethrin (12.1%) compared to the other three populations (p ≤ 0.001). Exposure to DDT (organochlorine) resulted in mortalities ranging from 67 to 95% supporting a moderate resistance. High tolerance to DDT was still evident in Njombe-Penja and Mibellon. The mortality rates were 67% and 79%, respectively, a marked difference from the other two sites, which had a mortality rate of approximately 95%. Unlike the full susceptibility against bendiocarb observed in Mibellon, the three other populations were resistant especially in Njombe-Penja which exhibited a high tolerance with 49% mosquitoes surviving 24 h after exposure. This was not the case for propoxur as mosquitoes from the four locations showed probable resistance (90–97% mortality) (Fig. 2B). Overall, the mosquito population from Njombe-Penja showed the highest resistance, with a high tolerance to three major insecticide classes: pyrethroids, organochlorines, and carbamates. This resistance is particularly notable given the industrial banana plantation in the area. In contrast, all mosquito populations were fully susceptible (100% mortality) to the organophosphates malathion and pirimiphos-methyl (Fig. 2B).

### Intensity of pyrethroid resistance

Bioassays conducted using increase concentration of permethrin, deltamethrin and alpha-cypermethrin at 5 × and 10 × DCs (Fig. 2C) showed a variation in resistance intensity according to geographical location and agricultural practices. In the South for example, in Njombe, a banana plantain cultivation farm, 20% of the mosquito survived at permethrin 10 × dose, highlighting high intensity of resistance in this location. This contrasts with Elende where only less than 5% of mosquito survived at the same dose. Intriguingly, in the Northern, while high intensity of resistance was recorded in Mibellon with roughly 94% mortality at 10x, a moderate intensity (100% mortality at 5 × DC) was noticed in Gounougou, a rice field area. Regarding deltamethrin and alphacypermethrin, a high level of resistance was observed in Mibellon at 10 × for both insecticides with 84.9% and 81.9% mortality rates recorded, respectively (Fig. 2C). This was followed by similar trend in Gounougou, where 90% and 82% of the mosquito died after exposure to deltamethrin and alphacypermethrin at 10 × dose, respectively. This strength of resistance was different in Elende populations with mortalities of 96.7% at deltamethrin 10 × and 94.1% at alphacypermethrin 5x. The results collectively indicate an escalation of pyrethroid resistance across all four localities.

### Correlation between pyrethroid resistance and sporozoite infection rate

In order to correlate epidemiological and entomological data, the trend between sporozoite rate and the level of pyrethroid resistance was investigated. The infection rates vary across the study sites with no clear association with phenotypic resistance. For example, mosquito from the peri-urban area Elende shows a high sporozoite infection rate of 16.5% indicating a high level of malaria transmission (Table 1). This combines with high resistance intensity to pyrethroids, with a mortality rate ranging from 95–96.7% may likely contribute to the high infection rate. Mosquitoes from Mibellon exhibit a lower but still notable sporozoite infection rate of 5.4%. While lower than that of Elende’s infection rate, the high resistance intensity was even more pronounced in Mibellon with mortality ranging from 81–93%. In contrast, no sporozoite infection was detected in both wild mosquito from Njombe-Penja and Gounougou suggesting very low or no current malaria transmission at the time of sample collection. However, the entomological data is alarming with mortality of ≤ 80% in Njombe-Penja and ≤ 90% in Gounougou at 10 time the diagnostic concentration (Table 1).

**Table 1:** Sporozoite infection rate and pyrethroid resistance

Locality	Sporozoite infection rate	Mortality rate with Pyrethroids 1x (%)	Resistance intensity 10x (%)	Interpretation
Elende	16.5% (14/85)	3.9–70.6	95.8–96.7	High intensity resistance and high malaria transmission
Mibellon	5.4% (5/92)	18–38.6	81.9–93.9	High intensity resistance and moderate malaria transmission
Njombe-Penja	0% (0/92)	12.1–18.2	80.4	High intensity resistance with no detectable malaria transmission
Gounougou	0% (0/127)	40.0–75.0	82.0–90.0	High intensity resistance with no detectable malaria transmission

### Synergy test

Pre-exposure of *Anopheles* mosquitoes to PBO demonstrated a substantial increase in susceptibility (p < 0.001) for all pyrethroids tested in all mosquito populations indicating the implication of cytochrome P450s enzymes as the main route of resistance (Table 2). Similarly, PBO pre-exposure yielded substantial mortality as compared to bendiocarb alone (p < 0.001). However, the partial recovery of susceptibility after pre-exposure to PBO particularly in Mibellon suggests the contribution of other resistance mechanisms beyond P450s. The same trend was observed towards bendiocarb in Njombe-Penja and Gounougou after PBO synergism assay (Table 2). No mortality was observed in control mosquitoes exposed to PBO alone and non-impregnated paper.

**Table 2:** Effect of PBO pre-exposure on the susceptibility profile of mosquito to pyrethroids and bendiocarb

Localities	Insecticide	Insecticide alone Mortality rate (%)	PBO + Insecticide Mortality rate (%)	*P*-value	**95% CI**
Elende	Permethrin 1x	70.6 ± 5.21	97.1 ± 1.8	< 0.0001	16.0–37.5
	Deltamethrin 1x	54.0 ± 3.5	100.0	< 0.0001	35.9–55.9
	Alphacypermethrin 1x	16.3 ± 4.5	100.0	< 0.0001	74.8–89.4
Njombe-Penja	Permethrin 1x	18.2 ± 5.8	92.9 ± 1.5	< 0.0001	63.6–81.9
	Bendiocarb 1x	50.9 ± 12.5	93.0	< 0.0001	28.1–55.6
Gounougou	Permethrin 1x	74.0 ± 16.2	100.0	< 0.0001	17.8–34.6
	Deltamethrin	33.0 ± 3.7	100.0	< 0.0001	52.5–78.3
	Alphacypermethrin 1x	45.0 ± 6.3	100.0	< 0.0001	44.2–65.0
	Bendiocarb 1x	70.8	93.7	< 0.0001	23.8–52.2
Mibellon	Permethrin 1x	38.6 ± 9.7	92.8 ± 7.1	< 0.0001	41.1–64.4
	Deltamethrin 1x	35.3 ± 8.4	90.6 ± 2.4	< 0.0001	43.4–64.1
	Alphacypermethrin 1x	18.1 ± 3.1	95.2 ± 4.7	< 0.0001	63.6–82.5

No mortality was recorded in both controls: untreated paper and PBO alone. *CI* Confidence Interval and *PBO* Piperonyl butoxide

### Cone bioassays

The results of bio-efficacy of various bed nets are reported in Fig. 2D. Across all the sites, a tremendous loss of efficacy was observed in pyrethroid-only nets with 0–17% mortality recorded with Olyset, PermaNet 2.0, Interceptor and DuraNet. For the Dual active ingredient net Royal Guard, optimal efficacy was obtained in Gounougou while moderate efficacy was noted in Mibellon. In contrast, PBO-based nets Olyset Plus and PermaNet 3.0 roof showed an optimal efficacy across the study sites with mortality ranging from 82–100%. This increase of mortality with PBO nets compared to pyrethroids-only indicates a contribution of P450 enzymes to the reduce efficacy. No mortality was noted in mosquito exposed to untreated net.

### Distribution and spatio-temporal variation of resistant markers in field population of *An. funestus*

**Fig. 3:**
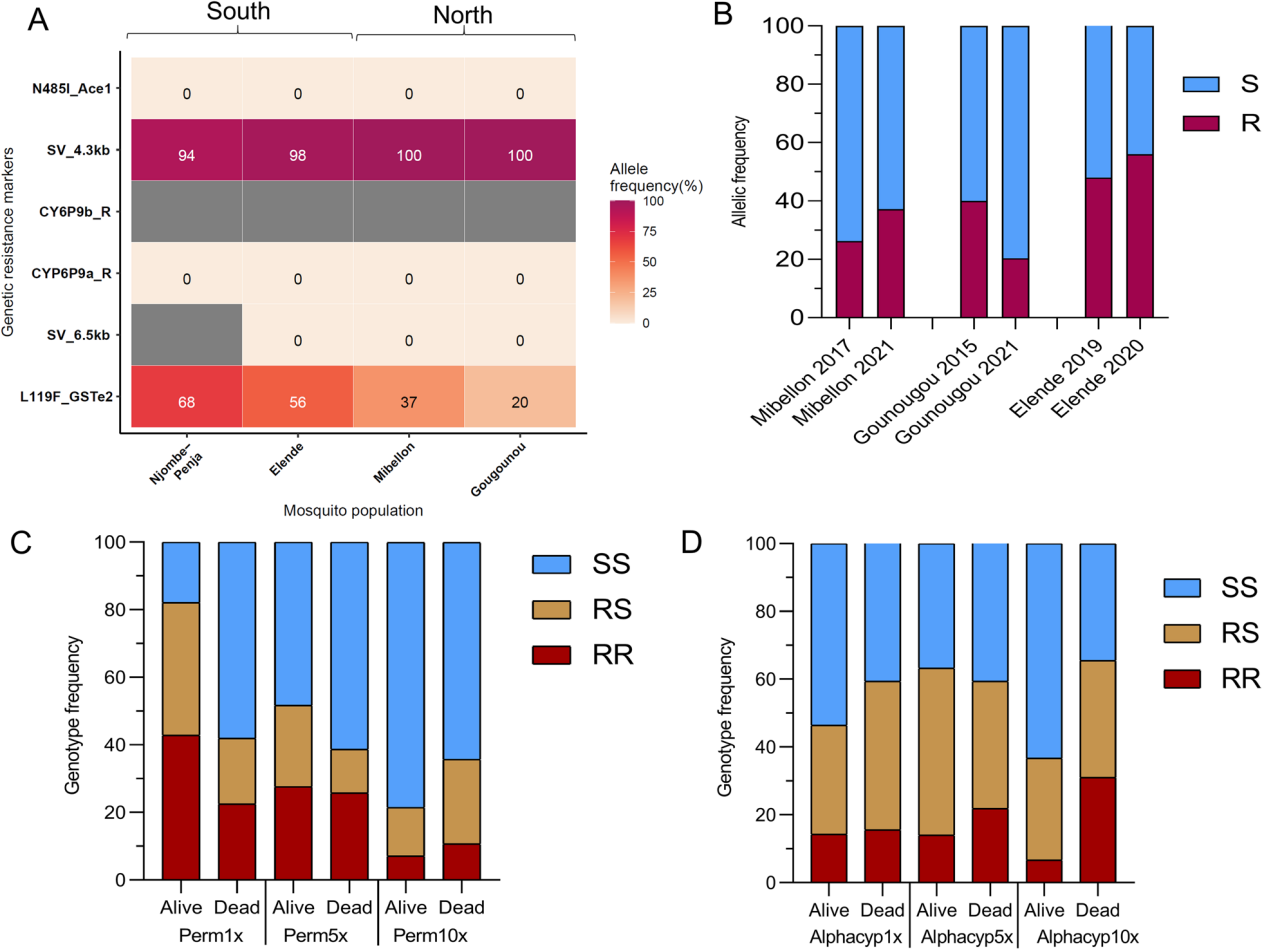
Distribution of known genetic resistant markers in *An. funestus*: **A** Distribution of allele frequency of key resistance markers *CYP6P9a/b*; 6.5 kb and 4.3 kb structural variants, the L119F-*GSTe2* and N485I *-Ace1* in field mosquito in the south-north transect, the grey represents samples that failed to amplify. **B** Temporal assessment of the allele frequency of the L119F-*GSTe2* resistant marker, **C** Genotypes distribution of the L119F-*GSTe2* between alive and dead mosquitoes after exposure to permethrin and **D** to alpha-cypermethrin at 1x, 5 × and 10 × DCs in Mibellon. Perm, permethrin; Alphacyp, Alphacypermethrin

Different resistant markers known to confer resistance to bendiocarb, pyrethroids and DDT were genotyped among a subset of 35 to 50 wild individual *Anopheles* mosquitoes from each locality (Fig. 3A). The N485I-*ace1* maker associated with bendiocarb resistance in southern Africa was not detected (100% SS) in the mosquito’s populations from Cameroon. The *CYP6P9a_R* allele and its enhancer 6.5 kb structural variant (SV) conferring resistance to pyrethroids were completely absent, however in Njombe the SV-6.5 kb failed to amplify (Fig. 3A). In contrast, the *CYP6P9b-R* allele failed to amplify in all sites probably due to the SV-4.3 kb which was fixed. Indeed, this insertion is within the promoter region of the *CYP6P9b* gene where the primers for the RLFP assay [28] are supposed to bind. There was a decrease in allele frequency for the L119F-GSTe2 mutation from far south to far north (68% vs 20%, p < 0.0001, χ^2^ = 23.1, 95% CI = 29–62.2%). Indeed, the frequency of the L119F-*GSTe2* mutation was significantly higher in the southern part, Njombe (68%) and Elende (56%) as compared to the northern localities, 37% and 20% in Mibellon and Gounougou, respectively. Conversely, the 4.3 kb SV was almost fixed (94–100%) across the four localities (Fig. 3A).

Temporal analysis of the L119F-*GSTe2* allele (Fig. 3B) showed a slightly increase in frequency in both Mibellon and Elende populations, albeit this was not significant (p > 0.1). Unexpectedly, in Gounougou this resistant allele decreased significantly from 40% in 2015 to 20.4% in 2021 (p = 0.02).

### Association between resistance markers and intensification of pyrethroids resistance

The L119F-*GSTe2* genotyping performed in 35 dead and 35 alive F_1_ progenies from Mibellon after exposure to permethrin and alpha-cypermethrin at 1x, 5x, 10 × DCs revealed that homozygote mosquitoes 119F/F (RR) significantly survived exposure to permethrin 1 × more than homozygote L/L119 (SS) (OR = 6.02, CI 2.9–12.5, *p* < 0.0001) (Fig. 3C). Similarly, heterozygotes (L119F) survived more to permethrin 1 × than those with the L119 (SS) (OR = 6.6, CI 3.1–14.1, *p* < 0.0001). However, no difference was noted between mosquitoes 119F/F homozygotes (RR) and heterozygotes L119F (RS) (OR = 0.9, CI 0.4–1.9, *p* = 0.8), suggesting that carrying a single resistant allele is sufficient in conferring resistant to permethrin 1 × and there is no additive effect (119F(R) versus L119 (S): OR = 3.5, CI 1.9–6.2, p < 0.0001) (Fig. 3C). No difference in genotype/allele distribution was observed between dead and alive to permethrin 5x (p > 0.1) and 10x (p > 0.1) DCs respectively (Fig. 3C) indicating that the L119F-GSTe2 drives resistance to permethrin 1 × but may have limited impact on resistance escalation. No association was found in the ability to survive alpha-cypermethrin 1 × and 5 × for all the resistant genotypes and allele (Fig. 3D). Surprisingly, in alpha-cypermethrin 10x, the homozygote resistants (RR) were significantly present among the dead compared to the alive (p < 0.01), potentially indicating a negative correlation between this mutation and resistance to high doses of alpha-cypermethrin (Fig. 3D). No further investigation was conducted in other localities as other genetic markers were either fixed or absent.

### Transcription profiling of detoxification genes

**Fig. 4:**
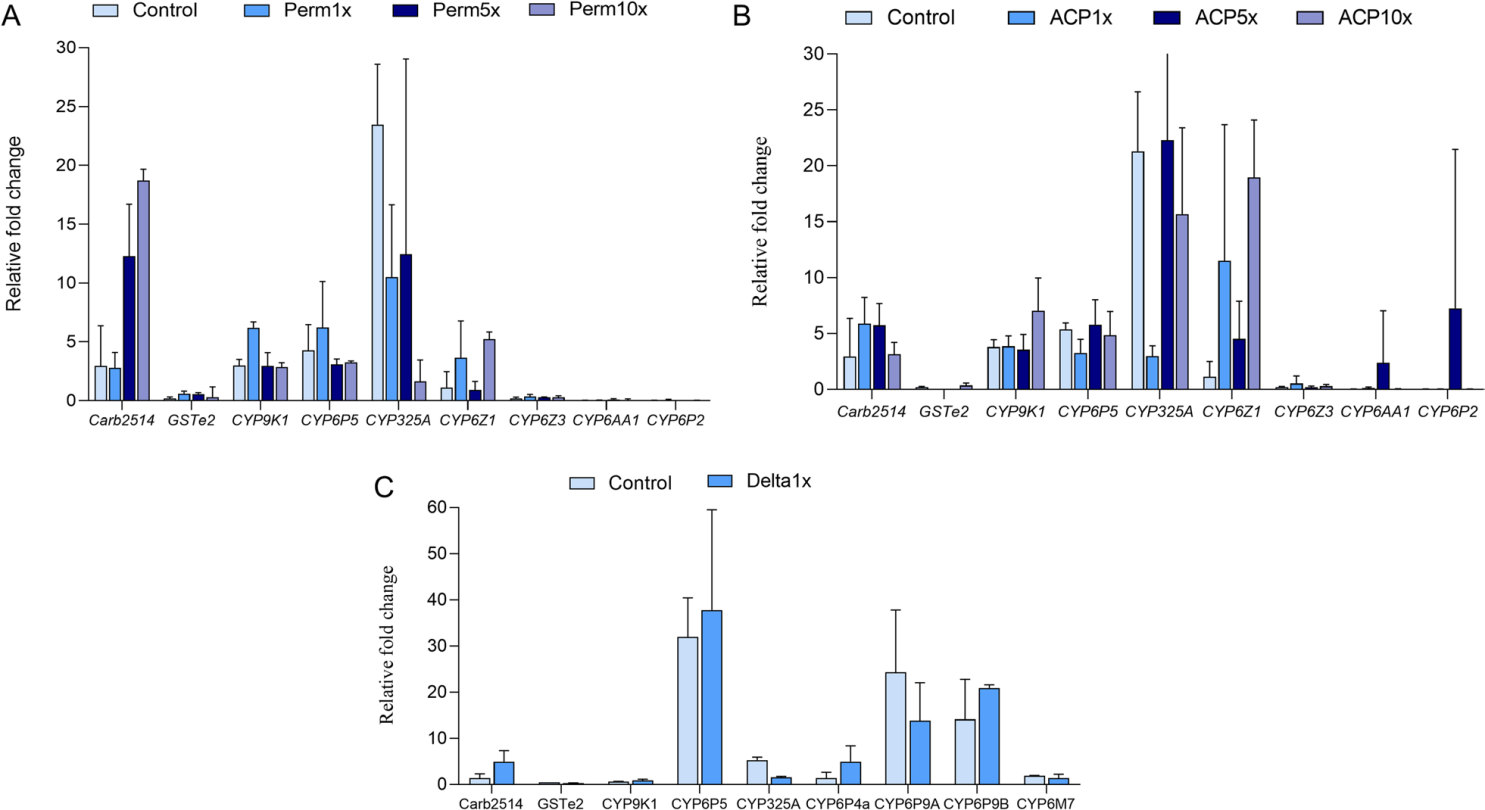
Differential gene expression of detoxification enzymes in *An. funestus* mosquitoes relative to the susceptible laboratory strain FANG. **A** Permethrin and **B** Alphacypermethrin in control (unexposed), 1 × , 5 × and 10 × DCs mosquito from Mibellon. **C** Control and Deltamethrin 1 × mosquito from Gounougou. Error bars represent standard error of the mean at 95% confidence interval. *Perm* permethrin; *ACP* alphacypermethrin; *Delta* deltamethrin

In Mibellon, mosquito surviving permethrin1x showed overexpression of *Carb2514, CYP6Z1 CYP9K1, CYP6P5*, *CYP325A* genes with fold changes (FCs) of 2.8, 3.7, 6.2, 6.2 and 10.5 respectively (Fig. 4A). Only *CYP9K1* gene showed an induced expression pattern in exposed mosquitoes compared to the unexposed (control) (p = 0.003), meanwhile other genes seem to be constitutively expressed (resistant versus unexposed, p > 0.05). Regarding alpha-cypermethrin 1x, *Carb2514* (5.9-fold), the P450s *CYP9K1* (3.9-fold), *CYP6P5* (3.3-fold), *CYP325A* (3-fold) and *CYP6Z1* (11.5-fold) were up-regulated compared to the susceptible lab strain FANG (Fig. 4B). Overall, the change in gene expression did not vary significantly between mosquito exposed to different diagnostic doses of pyrethroids.

In mosquito from Gounougou, *CYP6P5* (FC: 37.8) was by far the most overexpressed gene followed by *CYP6P9a* (24.3-fold), *CYP6P9b* (20.9-fold) and *Carb2514* (4.9-fold) with weak change in gene expression between deltamethrin resistant and control (Fig. 4C).

## Discussion

The rise of insecticide resistance in malaria vectors is threatening the efficacy of vector control tools. In this study, the phenotypic and genotypic status of *An. funestus* from four distinct geographical locations encompassing the south-north transect Cameroon were extensively characterised to efficiently guide subnational tailoring selection of control interventions in the country. Findings revealed high intensity of pyrethroid resistance across the studies sites and the restriction of strong bendiocarb resistance in one locality from the southern. Up-regulation of several metabolic enzymes coupled with the L119F-*GSTe2* resistant allele were marked in pyrethroid resistant mosquitoes.

Across the four locations, resistance was noted to three classes of insecticide including the organochlorine, pyrethroids and carbamate resistance restricted in two sites. However, full susceptibility was noticed for the organophosphate (malathion and pirimiphos-methyl) indicating that these insecticides could be the best option for IRS in these areas to tackle the rise of pyrethroid resistance. However, the strong bendiocarb resistance noticed in Njombe-Penja requires more investigation using resistance intensity assays and assessment of its impact on control tools and possibly its origin as this insecticide is not used in the public health sector. The high resistance intensity to both type I and type II pyrethroids (less than 93% mortality recorded at 10 × DC) confirm the aggravation of resistance in *An. funestus* in line with the findings from southern Mozambique [8] and Uganda [3]. Similar evidence of pyrethroid resistance escalation has been reported in *An. gambiae* across Cameroon [15, 16, 19, 20]. The factors selecting for this increase resistance in the malaria vector may result from the scale-up of bed nets since 2017 in the studied region reaching coverage and usage rates of 81.8% and 80.1% respectively [14] and the extensive use of pesticides in agriculture [19, 22]. For instance, survey in the areas observed mainly pyrethroid-only nets such as PermaNet 2.0 and Olyset [30] and agriculture insecticides included three insecticides classes: pyrethroids, neonicotinoids, carbamates [12, 22, 31]. Therefore, including farmers in the resistance management strategies to prevent usage of the same ingredients for both public health and agriculture is indispensable. Another important source of selection pressure is the unregulated use of pyrethroid-based domestic insecticides for personal protection such as aerosols, coils and electric emanators that may contribute to resistance evolution in malaria vectors as observed in *Ae. aegypti* population from Brazil [46, 47]. Consequently, a multisectoral approach is required when designing vector control and resistance management strategies including public health, private sector and community leaders.

The high tolerance of the mosquito towards pyrethroid led to a dramatic inefficacy of pyrethroid only-nets corroborating similar trend with Olyset and PermaNet 2.0 in Cameroon [22, 23] and elsewhere in Africa [3, 8, 48]. In opposite, PBO-based nets (PermaNet 3.0 and Olyset Plus) showed an optimal efficacy (100% mortality) indicating that these nets remain good alternative to tackle this high pyrethroid resistant mosquitoes [49, 50] (https://apps.who.int/iris/bitstream/handle/10665/258939/WHO-HTM-GMP-2017.17-eng.pdf). This result agreed with synergist assay where a significant recovery of susceptibility (pyrethroid alone versus PBO + pyrethroid, *p* < 0.0001) was observed. However, the partial restoration of susceptibility suggests the contribution of other resistance mechanisms beyond P450s. The novel net Royal Guard (RG) had a low efficacy; this observation should be taken with cautions due to the inadequacy of cone assay in evaluating the performance [51] of this dual ingredient net which has pyriproxyfen, an insect growth regulator, in addition to alphacypermethrin. The findings support the current mass distribution campaign of bed nets in Cameroon since 2022 predominantly PBO and dual active ingredients nets. In the area of pyrethroid resistance, Interceptor G2 net (alphacypermethrin plus chlorfenapyr) has been shown to reduce the incidence of malaria cases outperforming Royal Guard, PBO and pyrethroid-only nets [52]. In addition, the change in biting behaviour of *An. funestus* from indoor to outdoor and earlier in the morning [27] noticed in Cameroon when people are awake from the bed nets underscores the urgent need to explore alternative interventions in the future including spatial repellent [53] and non-pyrethroid interventions [1] to reduce malaria transmission.

The high infection rate of 16.5% in the Elende population combined with high resistance intensity has significant epidemiological implication as pyrethroid-treated nets may be failing to effectively kill the mosquito increasing the risk of disease transmission. However, the present result could have been impacted by the overestimation due to ELISA, as previously reported [54]. This is exemplified by previous estimates of 4.6% and 8.7% infection rates [12, 27] which are 2 to 4 times less than the current result in this same location using PCR-based technique targeting the 18S rDNA gene [37, 38]. Similar trend of notable infection rate was observed in Mibellon population corroborating previous reports [26, 27] which may still pose a serious threat to malaria control. Altogether, there is a strong need for alternative vector control strategies such as Chlorfenapyr-based net (Interceptor G2) which have shown greater public health values in controlling pyrethroid-resistant mosquitoes [52, 55] as well as inhibiting *P. falciparum* development in the mosquito [56]. Future studies should consider all the vector population (especially in Gounougou where 34% of the mosquito collected were *An. gambiae*) when detecting the malaria parasite to capture the overall picture of sporozoite rate giving its implications for malaria transmission risk. This will be enhanced by using recent robust PCR-based technique which outperforms all other old techniques (targeting the circumsporozoite, cytochrome b and 18S rDNA genes) [57].

Overall, no clear differential pattern of resistance mechanism was observed, as the trend of genetic resistance markers was similar across the transect. The *CYP6P9a_R* and the 6.5 kb SV alleles were completely absent, these two resistant markers in addition to the *CYP6P9b_R* (that failed to amplify in this study), are the main drivers of pyrethroids resistance in Southern Africa [41, 42]. This might be due to restriction of gene flow between mosquito populations from Southern to Central Africa [58]. In contrast, the L119F-*GSTe2* was detected at a moderate to high frequency (20–68%) and explained resistance to DDT. Despite the fact that DDT is banned in Cameroon since 1960, the persistence of this allele across the studies sites could be explained by the cross-resistance with pyrethroids [26]. The frequency of the L119F-*GSTe2* is relatively higher than previously reported in Mibellon [22, 26] and Elende [16] contrasting with Gounougou population where this allele declined significantly over time [23], requiring further investigations. Furthermore, the L119F-*GSTe2* was associated to permethrin resistance only at the diagnostic dose (1x) indicating its minor contribution in permethrin resistance aggravation. Surprisingly, this L119F-*GSTe2* allele was significantly prevalent in dead mosquito as compared to alive at alphacypermethrin 10 × indicating a negative correlation which aligns with previous findings in Ghana [4] and warrant further investigations. Indeed, the *GSTe2* is more efficient in metabolizing DDT, and to a lesser extent type I pyrethroid than type II [59]. Contrary to the role of the N485I-*Ace*1 and the overexpression of the P450 *CYP6Z1* in driving bendiocarb resistance in Southern Africa [44], this study found a complete absence of the N485I-*Ace1* allele despite the observed strong tolerance to bendiocarb. However, gene expression patterns in bendiocarb-resistant mosquitoes were not examined in this study. Further research is necessary to unravel the molecular mechanisms underlying this rising resistance to help prevent the failure of future interventions using this insecticide.

The transcription profile showed a similar expression pattern between permethrin- and alphacypermethrin-resistant mosquitoes, with the genes *Carb2514, CYP9K1, CYP6P5, CYP325A* and *CYP6PZ1* being the most upregulated. These genes have previously been associated with pyrethroid resistance in *An. funestus* from the same locality in Cameroon (*CYP6P5, CYP325A, Carb2514*) [25, 41], in Uganda (*CYP9K1*) [3] and Malawi (*CYP6Z1*) [44]. However, the expression level did not vary significantly with increase concentration of pyrethroids [3, 20] revealing that other molecular drivers/mechanisms are contributing to this increasing tolerance to pyrethroids. The limitation of this study is that the impact of combined effect of genetic markers on the phenotype was not performed as they were either fixed or absent. However, the fixation of the *G454A-CYP9K1* allele, an efficient pyrethroid metaboliser detected recently [60] as well as the fixation of the 4.3 kb SV [28] combined with the overexpression of detoxification genes could explain the resistance phenotype observed in the present study. These observations combined with the lack of knock down resistance mechanism in this *An. funestus* population [23, 61] highlight the need of using whole genome sequencing technique to unveil the whole picture of the mechanisms underlying the escalation of pyrethroids resistance. This will provide useful information to the national malaria control programme to design and implement effective and targeted vector control interventions.

## Conclusion

This study confirms the escalation of pyrethroid resistance in *An. funestus* across the transect south-north Cameroon and the inefficacy of pyrethroid-only nets and highlights genes potentially implicated in the aggravation of insecticide resistance. The intense levels of pyrethroid resistance observed with mosquito surviving 10 times the diagnostic dose could lead to control failure and required particular attention when designing or planning to implement pyrethroid-based interventions especially in agricultural settings. To effectively manage the spread of insecticide resistance, it is crucial for Cameroon to conduct regular, nationwide monitoring of vector susceptibility to every class of public health insecticide. Urgent action should be taken to evaluate and implement alternative control measures to mitigate the challenge of pyrethroid resistance in the country.

## Data Availability

All the data supporting the findings of this article are included within the manuscript.

## Abbreviations

CI: Confidence interval
DC: Diagnostic concentration
DNA: Deoxyribonucleic acid
DDT: Dichlorodiphenyltrichloroethane
cDNA: Complementary deoxyribonucleic acid
FC: Fold change
GST: Glutathione S-Transferase
IRS: Indoor residual spraying
LLINs: Long lasting insecticidal nets
OR: Odds ratio
*PBO*: Piperonyl butoxide
PCR: Polymerase chain reaction
qRT-PCR: Quantitative reverse transcription polymerase chain reaction
RNA: Ribonucleic acid
*s.l.*: Sensu lato
*s.s.*: Sensu stricto
SV: Structural variant
WHO: World Health Organization
